# Genome‐wide analysis of bZIP gene family members in *Pleurotus ostreatus*, and potential roles of *PobZIP3* in development and the heat stress response

**DOI:** 10.1111/1751-7915.14413

**Published:** 2024-02-20

**Authors:** Yuanyuan Zhou, Zihao Li, Congtao Xu, Jinlong Pan, Haikang Li, Yi Zhou, Yajie Zou

**Affiliations:** ^1^ State Key Laboratory of Efficient Utilization of Arid and Semi‐arid ArableLand in Northern China Beijing China; ^2^ Institute of Agricultural Resources and Regional Planning Chinese Academy of Agricultural Sciences Beijing China

## Abstract

The basic leucine zipper (bZIP) transcription factor (TF) is widespread among eukaryotes and serves different roles in fungal processes including nutrient utilization, growth, stress responses and development. The oyster mushroom (*Pleurotus ostreatus*) is an important and widely cultivated edible mushroom worldwide; nevertheless, reports are lacking on the identification or function of bZIP gene family members in *P. ostreatus.* Herein, 11 bZIPs on 6 *P. ostreatus* chromosomes were systematically identified, which were classified into 3 types according to their protein sequences. Phylogenetic analysis of *Po*bZIPs with other fungal bZIPs indicated that *Po*bZIPs may have differentiated late. Cis‐regulatory element analysis revealed that at least one type of stress‐response‐related element was present on each bZIP promoter. RNA‐seq and RT‐qPCR analyses revealed that bZIP expression patterns were altered under heat stress and different developmental stages. We combined results from GST‐Pull‐down, EMSA and yeast two‐hybrid assays to screen a key heat stress‐responsive candidate gene *PobZIP3*. *PobZIP3* overexpression in *P. ostreatus* enhanced tolerance to high temperature and cultivation assays revealed that *PobZIP3* positively regulates the development of *P. ostreatus*. RNA‐seq analysis showed that *PobZIP3* plays a role in glucose metabolism pathways, antioxidant enzyme activity and sexual reproduction. These results may support future functional studies of oyster mushroom bZIP TFs.

## Graphical abstract

11 *Po*bZIPs were characterized and analysed, the *PobZIP3*‐*PoHSP100* interaction showed that *PobZIP3* is vital to the heat stress response. *PobZIP3* enhanced tolerance to high temperature, and positively regulated the development of *P. ostreatus*.

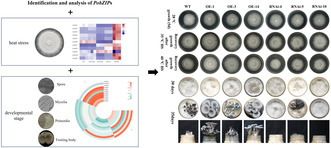

## INTRODUCTION

The oyster mushroom *Pleurotus ostreatus* has high nutritional and medicinal value and requires simple cultivation techniques (Dong et al., 2019; Hou et al., 2020; Jayasuriya et al., 2020; Qi et al., 2020; Xu et al., 2021). It is widely cultivated globally, and the Chinese Edible Fungi Association has reported that the yield of *P. ostreatus* in China of 6,113,400 tons in 2020 (Hou et al., 2021). Cultivation of *P. ostreatus* typically occurs in traditional greenhouses, which lack temperature and moisture control systems; therefore, the occurrence of extreme and sustained heat can lead to “spawn‐burning” and a predisposition to green mould infection, which eventually considerably affect *P. ostreatus* quality and yield (Hou et al., 2020; Zou et al., 2018).

Transcription factors (TFs) respond to changes in plant growth, developmental stage, and environmental stresses and subsequently regulate biological processes by controlling the expression of target genes (Baillo et al., 2019). TFs are one of the most important components of regulatory networks by binding to specific sequences of their target gene's promoter to regulate their expression (Hou et al., 2020). Based on its DNA‐binding domain, TFs are categorized into different TF families such as MYB, SBP, HB, DREB, NAC, bZIP, WRKY and AP2/EREBP (Wen et al., 2021). For example, thermal stress in rice induces the expression of the stress‐responsive TF *SNAC3*, and *SNAC3* overexpression can improve heat tolerance (Fang et al., 2015). Overexpression of the wheat TFs *WRKY1* and *WRKY33* enhanced drought and heat tolerance in *Arabidopsis thaliana* (He et al., 2016). Furthermore, in a 38°C heat treatment of *Dimocarpus longan* Lour, Zheng et al. found that nine *DlbZIP*s responded differently to varying durations of heat exposure (Zheng et al., 2020); moreover, overexpression of *TaOBF1‐5B*, one of the heat‐responsive *TabZIP* members, significantly improved recovery in *A. thaliana* after thermal stress compared to wild‐type (WT) (Samtani et al., 2022).

The bZIP family contains two regions: the basic region and the leucine zipper region, each with different functions (Zhang et al., 2020). The basic region comprises 18 amino acids, which form the conserved N‐X_7_‐R/K‐X_9_ motif that regulates nuclear localization and DNA binding (Wang, Wang, et al., 2020). The leucine zipper region mediates the homo‐ or hetero‐dimerization of bZIP TFs and includes several repeating motifs that contain leucine (or other hydrophobic amino acids including valine, isoleucine, methionine and phenylalanine) (Hu et al., 2016). bZIP family members have been found in animals, plants and microbes, and have been shown to play essential roles in both development and various stress responses (Xu et al., 2020). The number of bZIP genes has been found to vary among the different fungal species (Wen et al., 2021): 22 in *Magnaporthe oryzae* (Kong et al., 2015), 34 in *Coniothyrium minitans* (Xu et al., 2020), 26 in *Fusarium graminearum* (Son et al., 2011), 28 in *Ustilaginoidea virens* (Yin et al., 2017) and 26 in *Cytospora chrysosperma* (Wen et al., 2021). These bZIP TFs perform critical functions in fungal stress responses, growth and development, nutrient utilization and pathogenicity (Kong et al., 2015). For example, changes in the expression of the *CmbZIP* genes significantly alter the responses to abiotic stresses (cold, heat, NaCl, osmotic, oxidative, reductive and acidic), conidial development and mycoparasitism. Additionally, Δ*CmbZIP16* mutants showed a significantly reduced tolerance to oxidative stress (Xu et al., 2020); and there was a significantly reduced growth rate and pathogenicity, with an enhanced H_2_O_2_ sensitivity, in Δ*CcbZIP05* and Δ*CcbZIP23* mutants (Wen et al., 2021). *LtAP1*, a necrotrophic fungal bZIP that is a critical part of the stress response, also regulates the fungal ROS detoxification system and the plant defence response, which influences its pathogenicity (Wang, Zha, et al., 2020). bZIP TFs also exhibit a key function in the fungal thermal stress response. In pepper plants, research has shown that *CabZIP63* can modulate the expression of *CaWRKY40* and form a positive feedback circuit with it during the plant's response to *Ralstonia solanacearum* inoculation or heat and high moisture (Shen et al., 2016). Knockout of *AtfA*, one of the *Aspergillus fumigatus* bZIP TFs, increases the strain's sensitivity to both thermal and oxidative stress; furthermore, *AtfA* regulates some genes associated with stress protection such as *catA*, *dprA*, *scf1* and *conJ* (Hagiwara et al., 2014). However, bZIP TFs have not been identified in edible mushrooms; therefore, the response of edible fungal bZIPs to high temperature or their functions in growth and development was unknown.

bZIP family members can activate or inhibit the function of downstream targets by regulating their gene expression. The binding specificity and affinity of bZIP TFs with their target genes can be regulated through interactions with other proteins, phosphorylation modification and dimerization; in turn, the stability of the target can be further modified to impact its regulation of downstream physiological and biochemical processes (Schütze et al., 2008). Members of the plant bZlP TF family can bind to the ABRE (CCACGTGG), H‐box (CCTACC), G‐box (CACGTG), GLM (GTGAGTCAT), PB‐like (TGAAA4) and ACEs (ACGT) elements of downstream genes (Z et al., 2014). In rice, *Os*bZIP23 directly influences a range of genes related to stress responses, hormone signalling and developmental processes, such as *OsPP2C49*, which plays a role in ABA signalling regulation and *OsNCED4*, which regulates ABA synthesis (Zong et al., 2016). The maize bZIP gene *ZmbZIP4* regulates gene expression of numerous stress‐responsive genes such as *ZmLEA2*, *ZmRD20*, *ZmRD21*, *ZmRab18* and *ZmGEA6*, which regulate osmotic stress; *ZmNHX3*, *ZmHAK5* and *ZmKUP10*, which regulate sodium and potassium ion transport; *ZmOXS3*, which regulates oxidative stress; and *ZmHSFA2*, which regulates heat stress proteins (Ma et al., 2018). However, for the *P. ostreatus* bZIP genes (*PobZIP*s), the identification of the stress‐related genes with which they interact, the genes they regulate or the pathways in which they are involved remains unknown.

Herein, we performed a genome‐wide study to identify 11 bZIP genes from *P. ostreatus*, which we subsequently characterized, classified and analysed. Furthermore, we assessed the protein properties, phylogenies, motif distributions, genetic architectures and cis‐regulatory elements among these *Po*bZIP family members. Additionally, we investigated the expression patterns of these *PobZIP*s under heat stress and during different developmental stages. Subsequently, *Po*bZIP3 was found to interact with *Po*HSP100 based on yeast two‐hybrid, GST‐Pull‐down and EMSA assays; furthermore, heat stress treatment and cultivation assays of WT, overexpression (OE)‐*bZIP3* and RNAi interference (RNAi)‐*bZIP3 P. ostreatus* strains revealed that *PobZIP3* positively regulated the mycelium heat resistance and development. This study provided valuable information on the *Po*bZIP TFs, including the identification of some members that may be related to thermal stress, which provides support for future studies of other bZIP members and possible targets for molecular breeding of *P. ostreatus*.

## EXPERIMENTAL PROCEDURES

### Fungal strains and culture media

The *P. ostreatus* (CCMSSC 00389) is preserved in the National Mushroom Improvement Center of China. Potato‐dextrose agar (PDA) medium was used to culture mycelium. Luria‐Bertani (LB) medium containing kanamycin (100 μg/mL) and rifampin (50 μg/mL) was used to culture *Agrobacterium tumefaciens* GV3101, which was used to transform *P. ostreatus*. LB containing kanamycin (50 μg/mL) was used to culture the Trans 1‐T1 phage‐resistant chemically competent cells, which was used to construct and expand the plasmid. The OE and RNAi putative transformants were screened in complete yeast medium (CYM, 10 g maltose, 20 g dextrose, 2 g yeast extract, 2 g peptone, 0.5 g MgSO_4_∙7H_2_O, 4.6 g KH_2_PO_4_, 1 L water) containing 0.09 mg/mL hygromycin B and 0.3 mg/mL cefotaxime. The mushrooms were cultivated using a cottonseed hull medium (94% cottonseed hull, 5% wheat bran and 1% lime mixed with 65% water content). Next, 180 g of the mixture was packed into 270 mL cultivation bottles and sterilized at 126°C for 120 min.

### Heat treatment

Five different treatments—one control and four heat stress—were used to assess the heat stress response of *Po*bZIPs in the CCMSSC 00389 strain. For the control treatment (CK), cultures were incubated at 28°C for 5 days. For the heat stress treatments of 10 min (HS 10 min), 30 min (HS 30 min), 24 h (HS 24 h) and 48 h (HS 48 h), the cultures were incubated at 28°C for 5 days, then subjected to heat stress at 40°C for 10 min, 30 min, 24 h and 48 h, respectively. To assess the function of *PobZIP3* in the heat stress response of the CCMSSC 00389 strain, *PobZIP3* overexpression (OE‐*bZIP3*) and RNAi (RNAi‐*bZIP3*) strains were incubated at 28°C for 5 days, and subjected to heat stress at 35°C and 40°C for 48 h followed by recovery at 28°C for 4 days.

### Identification of *P. ostreatus* bZIP transcription factors

The bZIP domain (PF00170 and PF07716) was downloaded from the Pfam database (http://pfam.xfam.org/) (Wang, Wang, et al., 2020). HMMER 3.2.1 tools were used for the HMM search (Wheeler & Eddy, 2013) with default parameters based on the protein sequences of *P. ostreatus* (Qu et al., 2016). InterProScan (https://www.ebi.ac.uk/interpro/) and NCBI Conserved Domain Database (NCBI‐CDD, https://www.ncbi.nlm.nih.gov/cdd) were used to analyse the bZIP domain region of the identified potential sequences to verify the presence of the bZIP domain (IPR004827 and IPR046347) (Wen et al., 2021). Sequences that were confirmed as *Po*bZIPs were submitted to the NCBI database.

### Gene structure and protein analyses of the *bZIP* gene family

The intron and exon information from the genome was analysed to confirm gene structure. IPC tools (http://isoelectric.org/) were used to estimate the isoelectric point (pI) and molecular weight (MW) (Hou et al., 2020). BaCelLo (http://gpcr.biocomp.unibo.it/bacello/) was used to predict the subcellular localization (Pierleoni et al., 2006) and Expasy (https://web.expasy.org/protparam/) was used to predict the instability index, aliphatic index and grand average of hydropathicity (GRAVY) (Cash, 1999) of the *Po*bZIPs.

### Sequence alignment, motif analysis, chromosomal location and protein tertiary structure prediction of *bZIP* genes

TBtools was used to visualize the conserved structural domains in the NCBI‐CDD‐ and Pfam‐predicted sequences (Chen et al., 2020). MEGA6 and MUSCLE v3.8.1551 were used for multiple sequence alignment of the protein sequences of *Po*bZIPs (Wen et al., 2021). TBtools was used to display the position of the *PobZIP* family genes from the *P. ostreatus* annotation GFF3 file.

### Construction of the *bZIP* gene family phylogenetic tree

The bZIP sequences from 10 fungus species (Table S1) were aligned, and then MEGA6 was used to construct a phylogenetic tree based on the maximum likelihood (ML) method IQ‐TREE, which uses the best fitting model and branch support computed with 1000 UltraFast Bootstrap replicates (Trifinopoulos et al., 2016; Wen et al., 2021).

### Cis‐element analyses of *PobZIP* genes

TBtools was used to extract the sequences 2 kb upstream from the translation start site of the *PobZIP*s. These sequences were submitted to the PlantCARE tool (https://bioinformatics.psb.ugent.be/webtools/plantcare/html/) for cis‐acting regulatory element analysis (Lescot et al., 2002); after manually removing redundant results, TBtools was used to draw the graph.

### Total RNA extraction and expression analysis of *PobZIP*s

The E.Z.N.A.^®^ Fungal RNA Mini Kit (R6840‐01, OMEGA Bio‐tek, Inc., Norcross, GA, USA) was used to extract total RNA, and the HiScript 1st Strand cDNA Synthesis Kit (R211, Vazyme, Nanjing, China) was used to synthesize cDNA according to the manufacturers' protocols, which was then diluted 10‐fold for future use.

RT‐qPCR was performed to analyse the effect of heat stress on the expression levels of 11 genes (Wang et al., 2017) using beta‐tubulin as the reference gene (Hou et al., 2020), and the relative expression level of the *PobZIP*s was calculated using the 2^−ΔΔCt^ method (Livak & Schmittgen, 2001). The primer sequences used for RT‐qPCR are listed in Table S2.

### RNA‐seq and differential expression analysis

The expression patterns of the *PobZIP* genes at different developmental stages were determined by RNA‐sequencing (RNA‐seq). Mycelia, primordia, spores and fruiting bodies were collected and immediately frozen in liquid nitrogen, with three replicates for each sample. After extracting the RNA for RNA‐seq, the TruSeq Stranded mRNA LTSample Prep Kit (Illumina, San Diego, CA, USA) was used to construct the sequencing library and the Illumina HiSeq 2500 platform was used for sequencing. TBtools was used to construct the heatmap.

### Yeast two‐hybrid assay

The *PoHsp100* gene fragments were amplified from the cDNA of *P. ostreatus* using the primers shown in Table S2, and the fragments were inserted into the pGBKT7 vector. The *PobZIP* gene fragments were amplified and inserted into the pGADT7 vector, the fusion plasmids were transformed into Y2HGold yeast receptor cells, and the cells were coated in SD/‐Trp/‐Leu nutrient‐deficient medium and incubated at 30°C to observe the growth of colonies. The monoclonal was inoculated into 100 μL of sterilized water and dilutions were spotted onto SD/‐Trp/‐Leu/‐His/‐Ade/AbA/X‐α‐Gal screening medium and incubated at 30°C to observe the colour change of the colonies.

### GST pull down

The *PoHsp100* gene fragments were amplified from the cDNA of *P. ostreatus* using the primers shown in Table S2, and the fragments were inserted into the pD2P‐His vector. The *PobZIP3* gene fragments were amplified and inserted into the pD2P‐GFP vector, and the fusion proteins were expressed using the eukaryotic cell‐free protein expression system (Sigma‐Aldrich (Shanghai) Trading Co. Ltd.) and purified with His‐ or GFP‐tagged magnetic beads. The protein purity and concentration were measured using SDS‐PAGE and BSA kits (BIO‐RAD, Hercules, CA, USA). The *Po*bZIP proteins were then mixed individually with the *Po*Hsp100 protein and incubated overnight at 4°C and 360 rpm. The GFP‐tagged magnetic beads were then used to purify the mixed protein and western blot was used to detect the results of the protein interaction.

### EMSA

*Escherichia coli* BL21 (DE3) was used to express the His‐tagged *Po*bZIP3 fusion protein (His‐bZIP3), which was then purified using Novagen^®^ Ni‐IDA Resin (Merck, Darmstadt, Germany). Biotin was used to synthesize the tagged oligonucleotides. Unlabelled oligonucleotides were used as competitors. To create double‐stranded probes, equal amounts of the single‐strand complementary oligos were mixed and heated to 95°C for 2 min, and then gradually cooled to 25°C. The probe sequences are shown in Table S2. The LightShift™ Chemiluminescent EMSA Kit (Thermo Fisher Scientific, Waltham, MA, USA) was used for EMSA. Banding reaction solutions were incubated for 15 min at room temperature, the marked probe was added and the mixture was incubated for 25 min at 15°C. The protein–probe mixture was separated using a 6% (v/v) native polyacrylamide gel and then transferred to a nylon membrane. After cross‐linking under UV light, DNA on the membrane was detected using a Chemiluminescent Nucleic Acid Detection Module kit (Tanon, Shanghai, China).

### Plasmid construction and genetic transformation

First, the gene fragments were amplified from the *P. ostreatus* cDNA using the primers shown in Table S2. Next, they were inserted into the fungal OE and RNAi vectors using the Uniclone One Step Seamless Cloning Kit (Genesand Biotech, Beijing, China) (Hou et al., 2019, 2021). The vectors were transformed into *A. tumefaciens* GV3101, which was subsequently used to mediate mycelial *P. ostreatus* transformation (Hou et al., 2021; Lei et al., 2017, 2019). CYM medium was used to screen the putative transformants, which were confirmed using PCR and RT‐qPCR.

### RNA‐seq analysis

For RNA‐seq analysis, the WT, OE‐*bZIP3* and RNAi‐*bZIP3* strains were incubated at 28°C for 5 days and subjected to heat stress at 40°C for 48 h; the mycelia were then collected to extract total RNA. RNA integrity was evaluated using a 2100 Bioanalyzer (Agilent Technologies, Santa Clara, CA, USA) and construction and sequencing of the RNA libraries were performed by BGI (Wuhan, China). SOAPnuke (https://github.com/BGI‐flexlab/SOAPnuke) was used to filter the sequencing data and the Dr. Tom Multi‐omics Data Mining system (https://biosys.bgi.com) was subsequently used to perform data mining and analyse the filtered data. Bowtie2 (https://bowtie‐bio.sourceforge.net/bowtie2/index.shtml) was used to align the clean reads (including known and novel, and coding and noncoding transcripts) to the reference gene set. RSEM v1.3.1 (https://github.com/deweylab/RSEM) was used to calculate the expression level of the genes. Finally, DEGseq was used to analyse differential expression (*Q* value ≤0.05). The Phyper function, based on the hypergeometric test, was used to perform gene ontology (GO) (http://www.geneontology.org/) enrichment analysis of the annotated differentially expressed genes. A *Q*‐value ≤0.05 was applied to identify the significant terms and pathways.

## RESULTS

### Identification of *P. ostreatus* bZIP genes

In total, 11 putative sequences containing the bZIP domain, including the bZIP1 (PF00170) or bZIP2 (PF07716) domains, were identified. These 11 bZIP genes from *P. ostreatus* were named *PobZIP1* to *PobZIP11* and uploaded to GenBank with accession numbers OP921971–OP921981.

As shown in Table 1, the 11 *Po*bZIP proteins ranged from 169 aa (*Po*bZIP10) to 1264 aa (*Po*bZIP4) in length, 19.77 kDa (*Po*bZIP10) to 143.07 kDa (*Po*bZIP4) in theoretical molecular weight, 5.00 (*Po*bZIP8) to 9.78 (*Po*bZIP10) in predicted pI values, and contained 0 (*Po*bZIP5) to 12 (*Po*bZIP4) introns. According to the predicted subcellular locations, five proteins are in the nucleus, five in the endoplasmic reticulum and one in the cytoplasm. According to the Expasy results, the *Po*bZIP proteins are hydrophobic and unstable, based on their high Instability index values (>40), Aliphatic index values and negative GRAVY values.

**TABLE 1:** Genes encoding *PobZIP* transcription factor.

*PobZIP* number	Locus number	gDNA(bp)	cDNA(bp)	Intron	Protein length (aa)	bZIP domain position	MW (kDa)	pI	Subcellular location	Instability index (II)	Aliphatic index	GRAVY
*PobZIP1*	00389_003646‐RA	1876	1395	8	464	47–93	51.98	5.99	Nucleus	56.36	66.92	−0.820
*PobZIP2*	00389_003364‐RA	1712	1644	1	547	431–489	56.45	9.74	Endoplasmic reticulum	62.80	46.67	−0.885
*PobZIP3*	00389_001869‐RA	1877	1659	4	552	136–195	59.27	5.04	Endoplasmic reticulum	68.57	53.99	−0.697
*PobZIP4*	00389_002225‐RA	4922	3795	12	1264	765–833	143.07	5.96	Cytoplasmic	46.02	71.37	−0.674
*PobZIP5*	00389_004506‐RA	879	879	0	292	200–250	30.96	5.02	Endoplasmic reticulum	62.78	70.00	−0.585
*PobZIP6*	00389_005255‐RA	1736	1620	2	539	153–234	57.41	5.35	Endoplasmic reticulum	60.67	68.01	−0.428
*PobZIP7*	00389_004840‐RA	990	936	1	311	180–195	34.00	7.11	Nucleus	92.06	43.47	−1.309
*PobZIP8*	00389_007705‐RA	1632	1578	1	526	479–517	59.41	5.00	Endoplasmic reticulum	66.85	58.66	−0.762
*PobZIP9*	00389_007746‐RA	1210	888	2	295	157–205	31.66	6.78	Nucleus	59.15	60.71	−0.786
*PobZIP10*	00389_000139‐RA	668	510	2	169	41–54	19.77	9.78	Nucleus	60.31	65.92	−0.981
*PobZIP11*	00389_009634‐RA	3134	2559	3	852	348–363	90.65	5.71	Nucleus	60.79	60.54	−0.618

**FIGURE 1:**
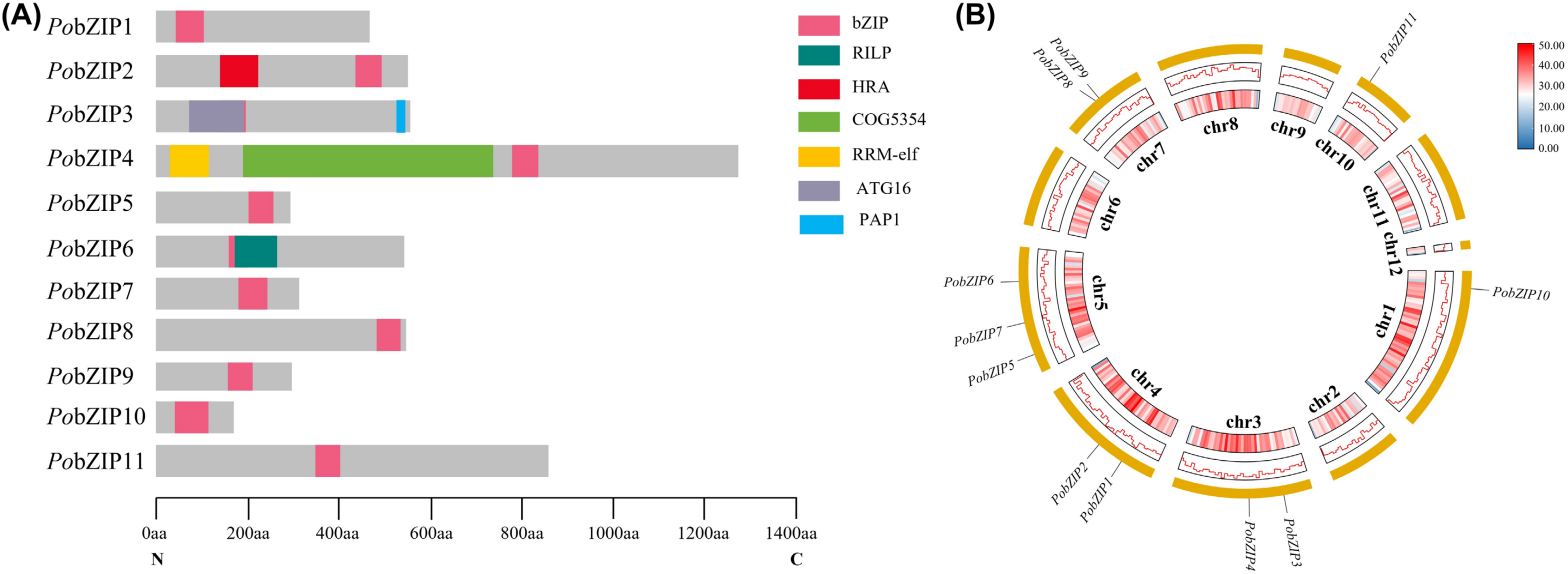
The conserved domain distributions and chromosomal locations of 11 *Po*bZIP family members in *Pleurotus ostreatus*. (A) Conserved domains of *Po*bZIPs. The domain organization of the bZIP proteins was determined from the Pfam and NCBI conserved domain databases. The X‐axis indicates the amino acid position from the N‐terminus to the C‐terminus. The grey rectangles indicate amino acid regions with unidentified domains and the different coloured rectangles indicate different conserved domains, as shown in the key. (B) Chromosomal locations of *PobZIP* genes. The inner and middle circles indicate the gene density of the chromosome and the outer circles indicate the position of the *PobZIP* genes on the chromosome.

Domain organization analysis showed that the conserved bZIP domains were found in the N‐terminus in four (36.36%) family members, in the middle in four (36.36%) family members, and in the C‐terminus in three (27.27%) family members (Figure 1A). In these 11 *Po*bZIPs, we also identified several other domains, which are highlighted in Figure 1A. For example, *Po*bZIP2, which contains bZIP domains in the C‐terminus, also contains Atf1‐HRA domains (PF11786), which was reported to be necessary and sufficient to activate recombination (Gao et al., 2008). Furthermore, in addition to its bZIP domain, *Po*bZIP3 also contains ATG16 and PAP1 domains, which were associated with macroautophagy and H_2_O_2_‐induced antioxidant gene transcription, respectively.

As shown in Figure 1B, the *P. ostreatus* bZIP genes were distributed on six separate chromosomes: Chromosome 1 (Chr1) and Chr10 each contained only one *PobZIP*; Chr3, Chr4 and Chr7 each contained two *PobZIP*s; and Chr5 contained three *PobZIP*s. The *PobZIP*s were not evenly distributed among the chromosomes, and no genes were preferentially distributed on specific chromosomes.

### Domain information of *PobZIP* genes in *P. ostreatus*

**FIGURE 2:**
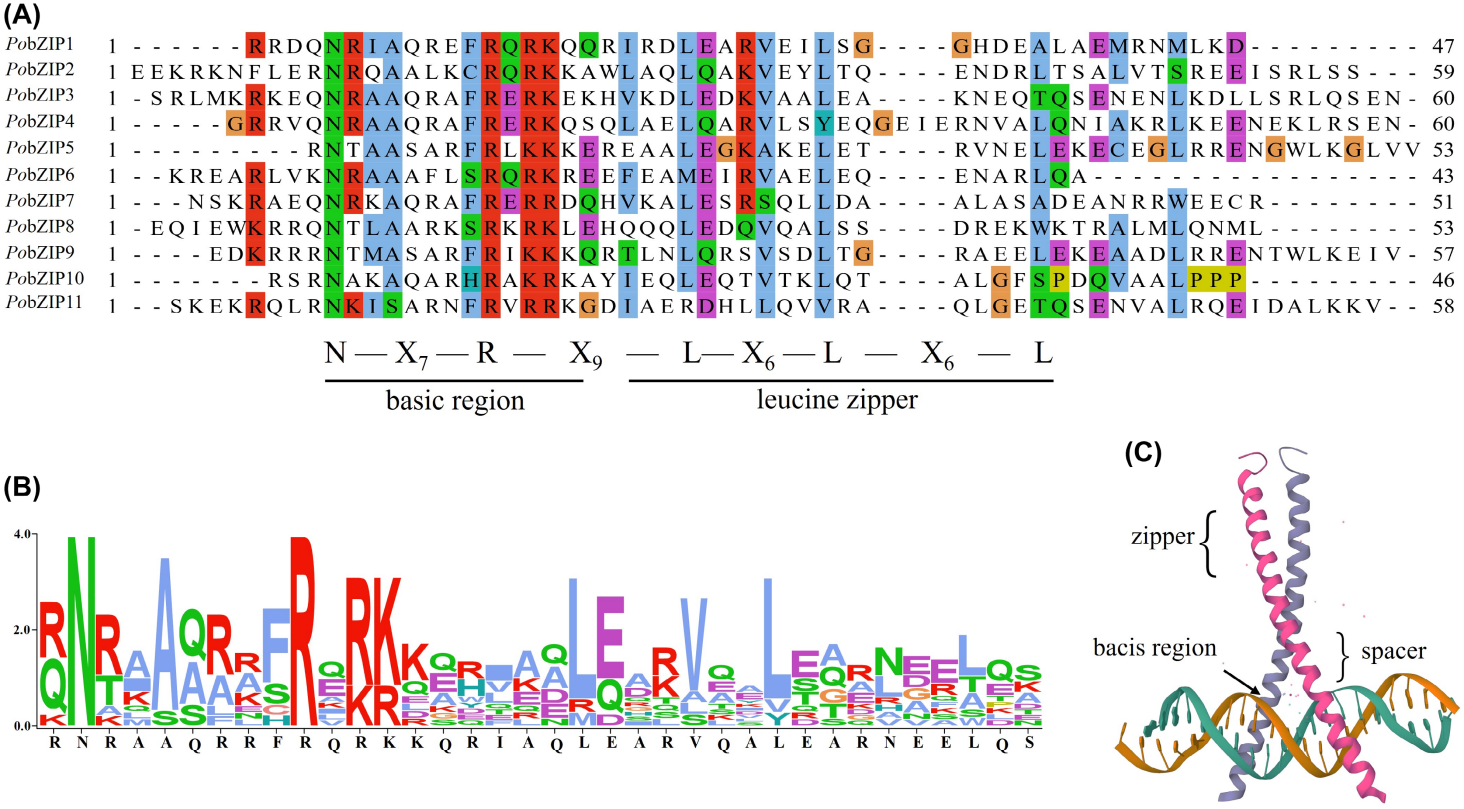
Multiple sequence alignment of the bZIP domain in 11 *Po*bZIP proteins in *Pleurotus ostreatus*. (A) Amino acid sequences of the bZIP domain were aligned using MUSCLE v3.8.1551 with minor modifications. (B) The sequence logo formed from the bZIP domain from the 11 *Po*bZIP proteins. The relative letter size of the amino acid residues indicates the frequency at which they appear at the same site. (C) Three‐dimensional bZIP protein structure.

The bZIP domain comprises an extremely conserved basic region characterized by the N‐X_7_‐R/K motif and a leucine zipper region characterized by a repeating motif in which leucine (or another hydrophobic amino acid) is repeated every seven amino acids. In order to study the *Po*bZIP domain characteristics, we performed a multiple amino acid sequence alignment, as shown in Figure 2A–C. These results showed an extremely conserved basic region in the *P. ostreatus* bZIP domains: All 11 *Po*bZIPs consistently showed the core asparagine (N) and arginine (R) residues with seven amino acids in between. However, the leucine zipper region was less conserved among the *Po*bZIPs, with other hydrophobic amino acids often replacing leucine in the following heptad repeat. In the first heptad repeat, leucine was replaced by methionine and arginine in *Po*bZIP6 and *Po*bZIP11, respectively; in the second heptad repeat, leucine was replaced by tyrosine and valine in *Po*bZIP4 and *Po*bZIP11, respectively; and in the third heptad repeat, which was even less well conserved, leucine was replaced by even more hydrophobic amino acids.

### Phylogenetic analysis

**FIGURE 3:**
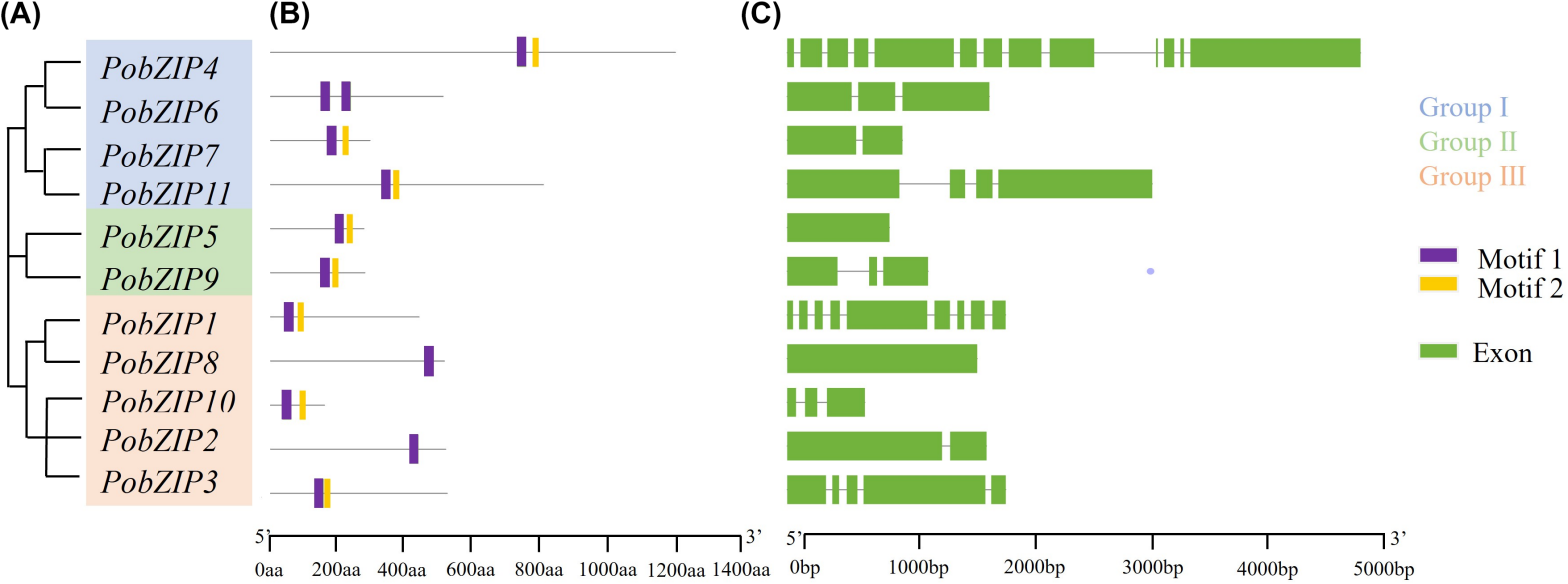
Phylogenetic analysis of 11 bZIP family members in *Pleurotus ostreatus*. (A) Phylogenetic tree based on the maximum likelihood (ML) method using IQ‐TREE with best fitting model and branch support computed with 1000 UltraFast Bootstrap replicates. The classification of different types of *Po*bZIPs (Groups I–III) is indicated with different colours, as shown in the key. (B) Motif composition of the *Po*bZIP proteins, obtained by using the MEME program. The grey line indicates the protein length and the coloured rectangles indicate different motifs, as shown in the key. (C) *PobZIP* gene structures. The exons are indicated by green rectangles and the introns are represented by the black lines connecting two exons.

A phylogenetic tree was constructed from the sequence alignment results, which showed that the 11 *Po*bZIPs were divided into three groups (I–III) (Figure 3A). The *PobZIP* genes exhibited a wide range in the number of introns, from 1 (*PobZIP2*, *PobZIP7* and *PobZIP8*) to 13 (*PobZIP4*); although some genes were clustered together, their motif positions (Figure 3B) and gene structures (Figure 3C) were dissimilar, suggesting that *PobZIP*s are highly variable.

**FIGURE 4:**
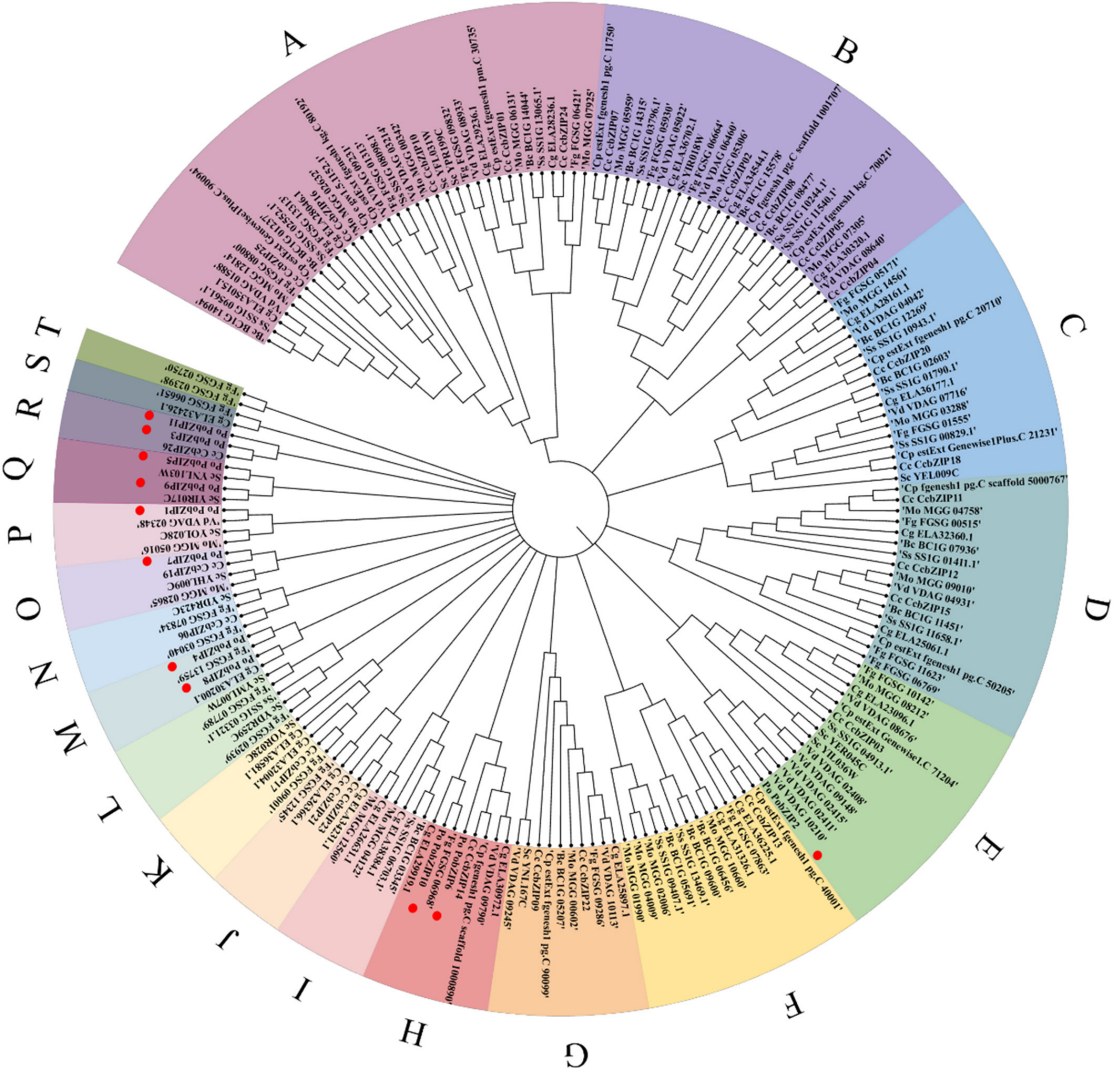
Phylogenetic analysis of bZIP genes in 10 fungal species. Different clades are shown in different colours, *PobZIP*s are marked with red dots. Bc: *Botrytis cinerea*; Cg: *Colletotrichum gloeosporioides*; Cp: *Cryphonectria parasitica*; Fg: *Fusarium graminearum*; Mo: *Magnaporthe oryzae*; Sc: *Saccharomyces cerevisiae*; Ss: *Sclerotinia sclerotiorum*; Vd: *Verticillium dahliae*; Cc: *Cytospora chrysosperma*; Po: *Pleurotus ostreatus*.

We explored the evolutionary relationship between other fungal bZIP genes and *PobZIP* genes based on our phylogenetic analysis (Figure 4 and Table S1). The 192 fungal bZIPs were classified into 20 clades, which were named from A to T. The 11 *PobZIP* genes were distributed among 7 clades, three *PobZIP*s were divided into two large clades, including *PobZIP2* (clade D) and *PobZIP6* and *PobZIP10* (clade H). The eight remaining *PobZIP*s were divided into five different small clades containing few bZIPs. *PobZIP3* and *PobZIP11* were sorted into clade R which also contains *CcbZIP26* that may have an important function in *C. chrysosperma* pathogenicity (Wen et al., 2021). The bZIP superfamily is derived from only one eukaryotic gene precursor that has evolved through numerous independent amplification events (Jindrich & Degnan, 2016). These results may indicate that bZIP TFs differentiated late in *P. ostreatus*.

### Cis‐element analysis

**FIGURE 5:**
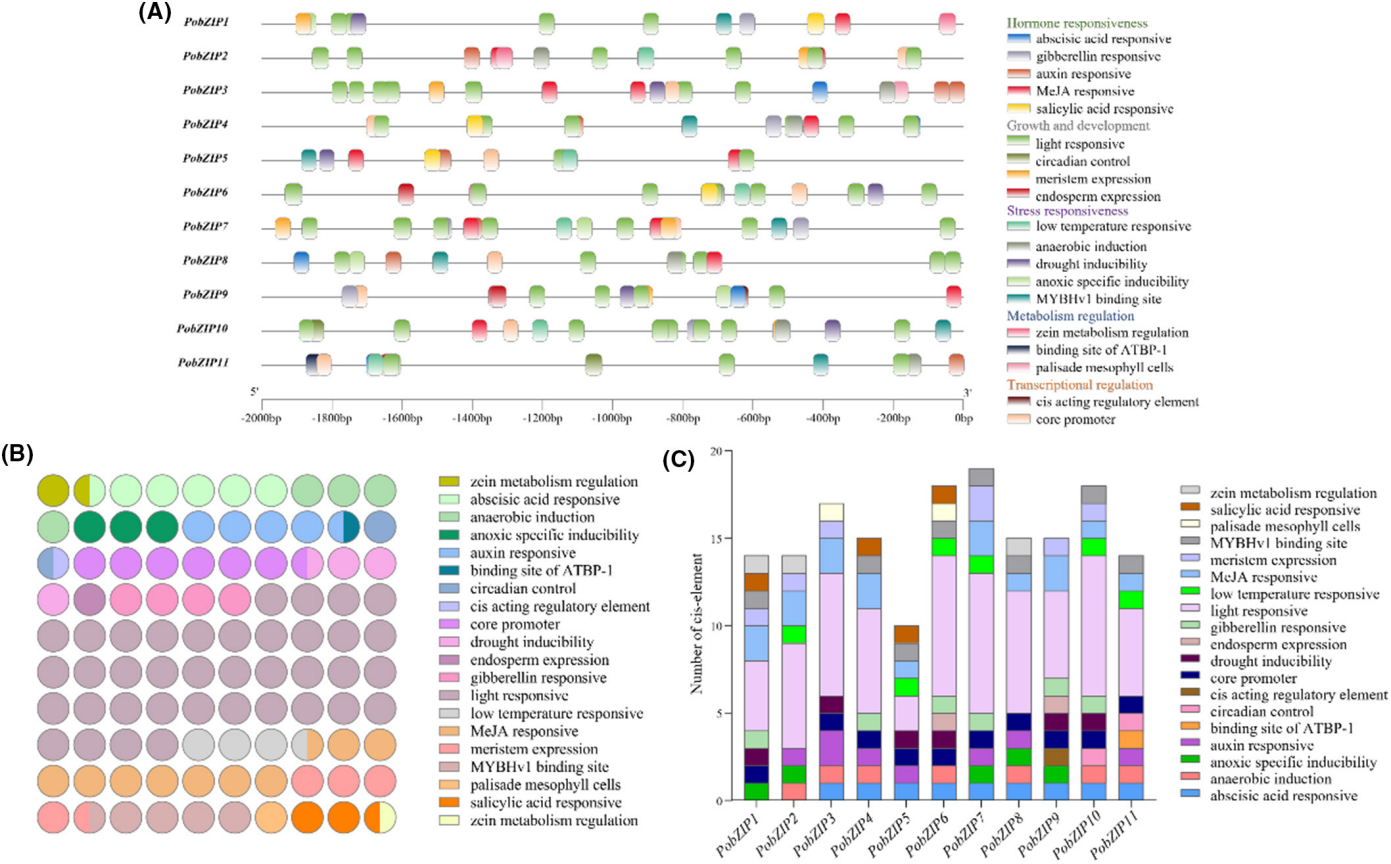
Predicted cis‐elements in the *PobZIP* promoters in *Pleurotus ostreatus*. (A) Cis‐regulatory elements in the promoters of the *PobZIP* genes within 2000 bp of the transcription start site. (B) Number of cis‐regulatory elements related to a specific function. Functions are indicated by different colours and the number of circles indicates the number of cis‐regulatory elements related to that function. (C) Number of cis‐regulatory elements within each *PobZIP* promoter. Functions are indicated by different colours.

Evaluating the cis‐acting elements within promoters is one of the keys to understanding the mechanism of gene regulation and predicting gene functions (Wang et al., 2021). We analysed cis‐elements within 2000 bp of the promoters to explore the possible biological roles of *PobZIP*s. Figure 5A shows the phenotype and frequency of 20 elements in the 11 *PobZIP* genes. These elements were categorized into five groups: hormone responsiveness, growth and development, stress responsiveness, metabolism regulation and transcriptional regulation. Overall, there were more elements related to growth and development and fewer elements related to metabolism regulation and transcriptional regulation (Figure 5B). Among the 20 components, the most abundant was the light‐responsive component in the growth and development group, which was distributed among all 11 *PobZIP* genes; moreover, the light‐responsive‐related elements accounted for the majority of components in the growth and development group (approximately 85%), with elements related to circadian control, meristem expression and endosperm expression making up the remaining 15% of the group. However, the circadian control‐related elements in the growth and development group was only present in *PobZIP10* and *PobZIP11*, indicating that the growth and development‐related *PobZIP* genes are preferentially light‐dependent. Each of the 11 *PobZIP* genes contained more than 10 cis‐acting elements (Figure 5C), with the number of cis‐acting elements ranging from 10 (*PobZIP5*) to 19 (*PobZIP7*). This variety in the different categories of these cis‐acting elements suggested that *PobZIP*s may be capable of responding to a variety of signalling pathways.

### *PobZIP* expression patterns undergoing heat stress

**FIGURE 6:**
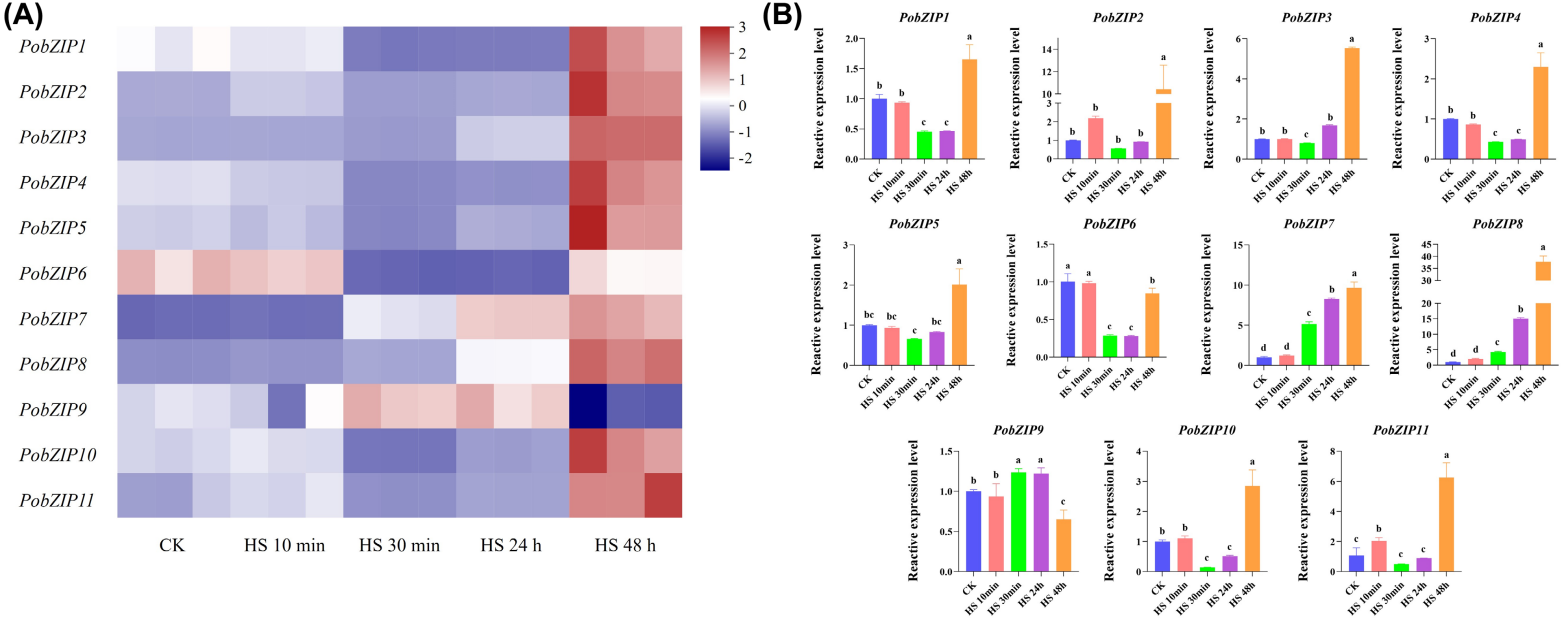
The RT‐qPCR gene expression patterns of *PobZIP*s in *Pleurotus ostreatus* under different durations of 40°C heat stress (HS) treatments. (A) The heatmap shows the relative expression of each *PobZIP*. The colour scale indicates the changes in expression levels, where red indicates increased and purple indicates decreased. (B) The expression level of each *PobZIP* relative to controls (CK) using beta‐tubulin as the reference gene. The different lower‐case letters indicate statistically significant differences between treatment groups.

The *PobZIP* gene expression profiles were determined based on RT‐qPCR data from samples subjected to 40°C heat stress treatments for various time intervals. As shown in Figure 6A, the *PobZIP* family genes showed different expression patterns under heat stress. At short‐term heat stress intervals (10–30 min), seven *PobZIP*s exhibited significantly different expression levels than the CK group, including three that were up‐regulated (*PobZIP2*, *PobZIP7* and *PobZIP8*) and four that were down‐regulated (*PobZIP1*, *PobZIP4*, *PobZIP6* and *PobZIP10*). The expression of *PobZIP2* after 10 min heat stress was 2‐fold higher than CK and the expression levels of *PobZIP7* and *PobZIP8* after 30 min heat stress were 5‐fold and 4‐fold higher than CK, respectively (Figure 6B). The expression of *PobZIP11* changed rapidly, with an initial 2‐fold up‐regulation after 10 min heat stress followed by a down‐regulation of 0.5 times CK after 30 min heat stress. At long‐term (48 h) heat stress intervals, the expression levels of eight *PobZIP*s (*PobZIP2*, *PobZIP3*, *PobZIP4*, *PobZIP5*, *PobZIP7*, *PobZIP8*, *PobZIP10* and *PobZIP11*) were significantly up‐regulated relative to the CK groups, ranging from 2‐fold to 37‐fold (Figure 6B). For example, *PobZIP3* expression was 5.5‐fold up‐regulated after 48 h heat stress relative to CK. However, *PobZIP4* expression varied with different heat stress times; it was down‐regulated after 30 min and 24 h heat stress intervals but up‐regulated after 48 h heat stress.

### *PobZIP* expression patterns during different developmental stages

**FIGURE 7:**
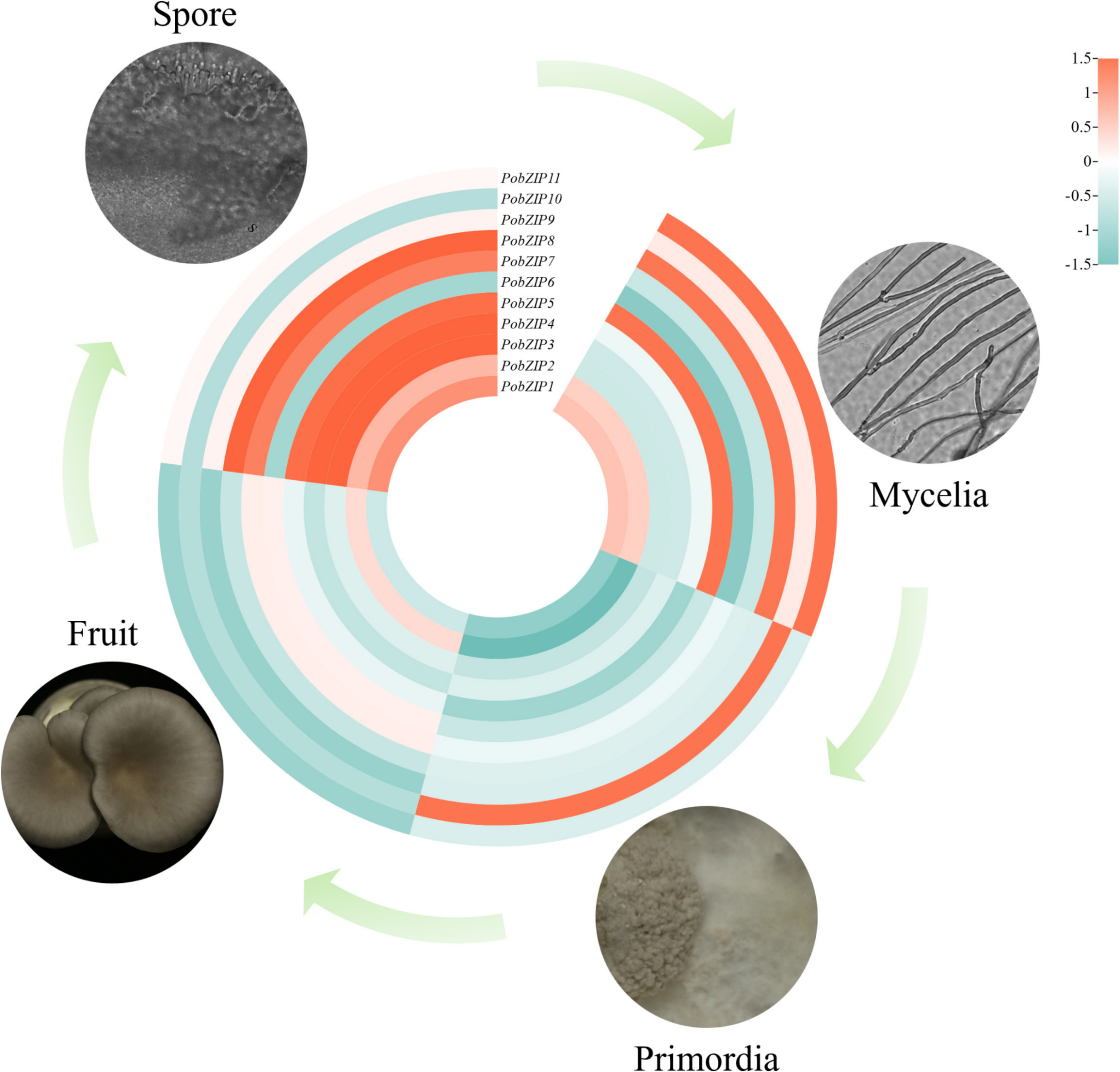
The expression patterns of *PobZIP*s during different developmental stages of *Pleurotus ostreatus*. The colour scale indicates the changes in expression levels.

We used RNA‐seq data to analyse the *PobZIP* gene expression profiles at different developmental stages. As shown in Figure 7, the expression patterns of the *PobZIP*s varied during the different stages of development. *PobZIP1* and *PobZIP4* were weakly expressed during the different developmental stages, whereas *PobZIP2* and *PobZIP11* were highly and stably expressed. *PobZIP5* expression during the mycelial stage was 3‐fold higher than during the primordial stage, while the expression of *PobZIP7* in the mycelial was 0.4 times that of the primordial stage. The expression levels of *PobZIP9* and *PobZIP10* in the primordial stage were 3 and 34 times higher than in the fruiting body, respectively, while the expression of *PobZIP5* in the primordial stage was 0.3 times that of the fruiting body. The expression levels of *PobZIP3*, *PobZIP4* and *PobZIP9* in spores were at least 3.5 times greater than in the fruiting body; specifically, *PobZIP3* expression was 3.65‐fold higher in the fruiting body than in the spore developmental stage. This variation in the expression of *PobZIP*s during the different growth stages may indicate that they are involved in growth and development‐related regulation.

### Interaction between *Po*bZIP3 and *Po*HSP100

**FIGURE 8:**
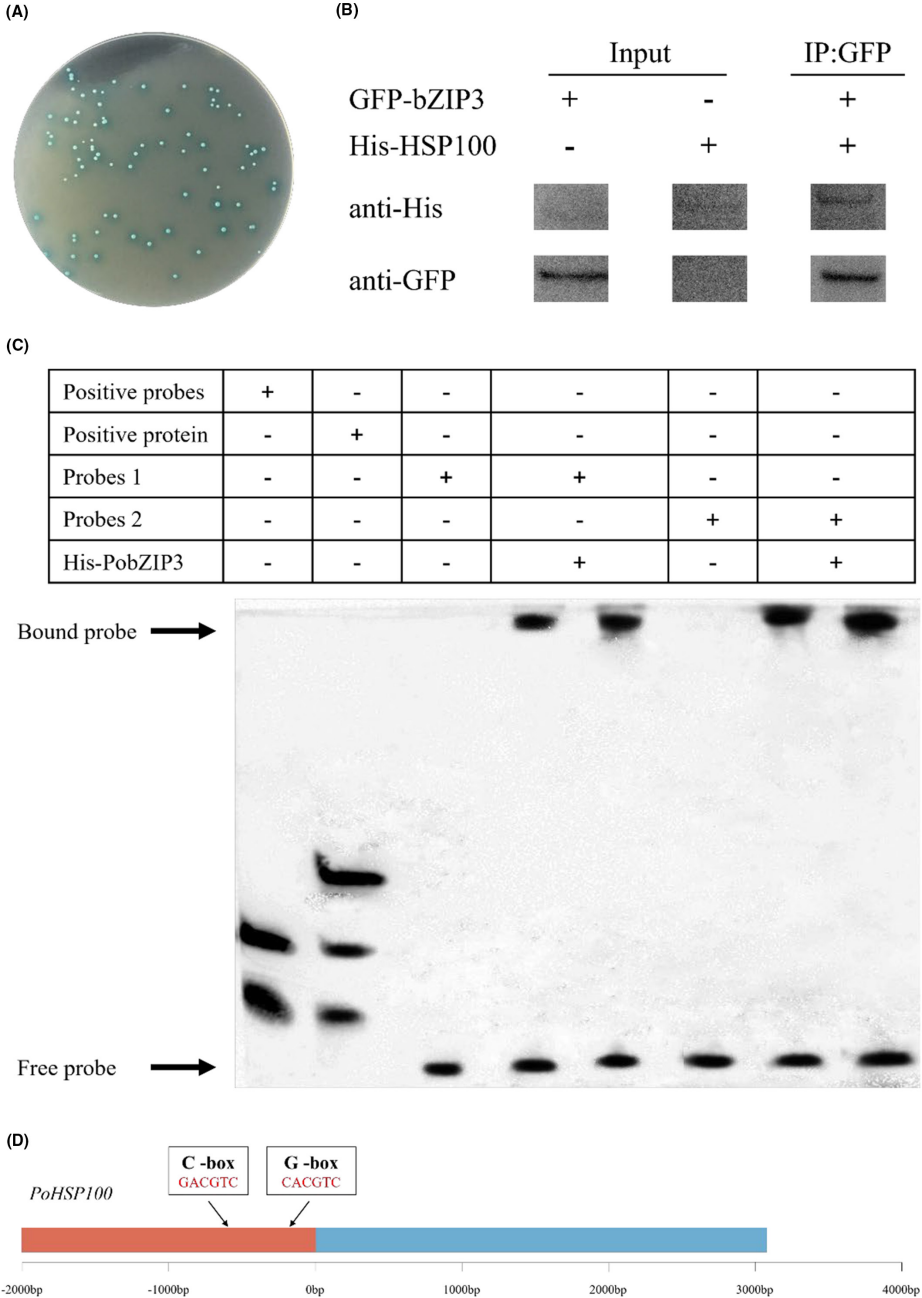
The protein *Po*bZIP3 interacts with *Po*HSP100 in *Pleurotus ostreatus*. (A) *Po*bZIP3 interacts with *Po*HSP100 in yeast based on blue colonies formed from a yeast two‐hybrid assay. (B) In vitro pull‐down assay showing the *Po*bZIP3–*Po*HSP100 interaction. Recombinant GFP‐bZIP3 proteins were immobilized on glutathione agarose beads and incubated with His‐HSP100 proteins. HSP100 was detected with anti‐His antibody. (C) *Po*bZIP3 binds to the C‐box and G‐box of the *PoHSP100* promoter. Probe 1 sequence: CTGGCGCACCGACGTCTCTTGA; Probe 2 sequence: CGAGTCTTCCCACGTGGCTTAG. (D) The location of C‐box and G‐box in the *PoHSP100* sequence.

In our previous research, we found that HSP100 is vital for heat resistance (Zou et al., 2018). To investigate whether *Po*bZIPs and *Po*HSP100 interact, we performed a yeast two‐hybrid assay and found that only yeast receptor cells co‐transformed with pGADT7‐HSP100 and pGBKT7‐bZIP3 vectors were able to produce blue colonies on SD/‐Ade‐His‐Leu‐Trp/X‐α‐Gal/AbA medium plates (Figure 8A), indicating that *Po*bZIP3 and *Po*HSP100 may interact.

To further confirm the interaction between *Po*bZIP3 and *Po*HSP100, pD2P‐His‐*HSP100* and pD2P‐GFP‐*bZIP3* vectors were constructed to express the fusion proteins His‐HSP100 and GFP‐bZIP3, respectively, and purified using their respective tags. The purified His‐HSP100 and GFP‐bZIP3 were incubated in vitro, pulled down with the GFP tag and detected using western blot. As shown in Figure 8B, when GFP‐bZIP3 was present, His‐HSP100 was pulled down and detected by the His antibody, indicating that the *Po*HSP100 and *Po*bZIP3 proteins interacted with each other in vitro.

DNA sequences that are able to bind to bZIP TFs contain relatively conserved ACGT core motifs, including the common G‐box (CACGTG), C‐box (CACGTC) and A‐box (TACGTA) (Baloglu et al., 2014; Jin et al., 2014). By analysing the sequence 2000 bp upstream of *PoHSP100*, we revealed the location of a G‐box and a C‐box at 579 bp and 188 bp upstream, respectively (Figure 8D). Therefore, we designed probes based on these two sequences and then performed EMSA with the purified proteins and probes, which revealed that *Po*bZIP3 bound to both sequences (Figure 8C).

### *Po*bZIP3 positively regulates the heat stress response in mycelial growth

**FIGURE 9:**
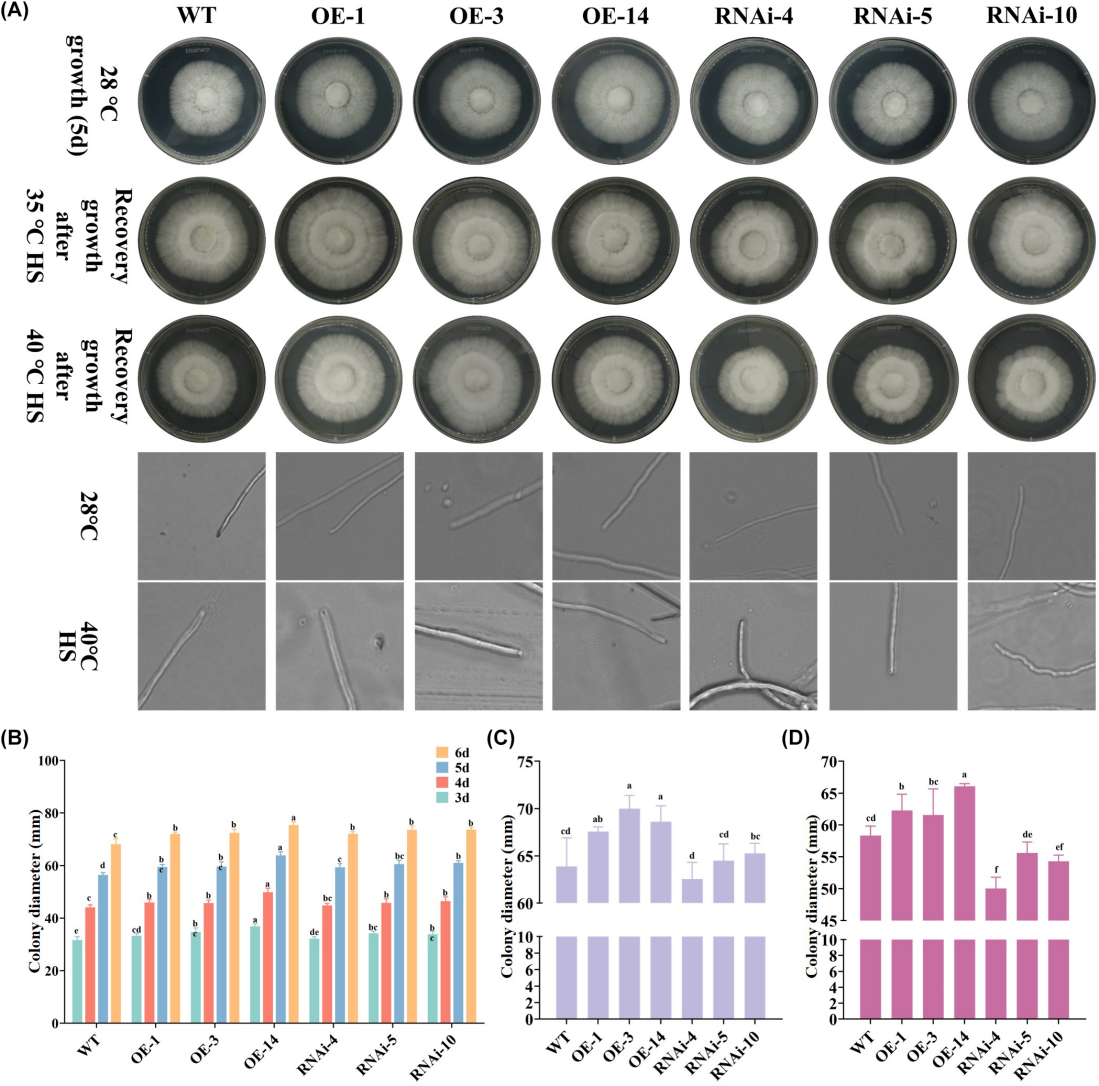
*PobZIP3* enhances mycelium growth recovery in *Pleurotus ostreatus* subjected to heat stress (HS). (A) Recovery of wild‐type (WT), overexpressed (OE)‐*PobZIP3* and RNAi‐*PobZIP3* strains after HS for 48 h, and hyphal branches in the tested strains cultured on PDA plates at 28°C and 40°C (scale bar = 50 μm). (B) Colony diameter of WT, OE‐*bZIP3* and RNAi‐*bZIP3* strains at 28°C. (C, D) Colony diameter of recovery growth when the WT, OE‐*bZIP3* and RNAi‐*bZIP3* strains subjected to (C) 35°C HS or (D) 40°C HS.

OE‐*bZIP3* and RNAi‐*bZIP3* plasmids were successfully constructed and transformed into *P. ostreatus* via *Agrobacterium*‐mediated transformation. The different strains (WT, OE‐*bZIP3*, RNAi‐*bZIP3*) were then cultured under various conditions to investigate the *PobZIP3* gene function in response to heat stress (Figure 9). When the strains were grown at 28°C, only the OE‐*bZIP3*‐14 strain showed a significantly higher growth rate than the other groups, while the growth rates of the other strains did not differ significantly (Figure 9A,B). After 5 days of growth recovery, the OE‐*bZIP* strains demonstrated a faster recovery rate with a complete colony edge relative to the WT strain, whereas the RNAi‐*bZIP* strains demonstrated a slower mycelial recovery rate with colony edge defects. In addition, the mycelium growth morphology was observed under the light microscope, which showed thicker mycelia of the OE‐*bZIP3* strains than those of the WT and RNAi‐*bZIP3* strains; furthermore, the mycelial RNAi‐*bZIP3* mutants became twisted after 40°C HS (Figure 9A,C,D). Therefore, the *bZIP* genes are actively involved in *P. ostreatus* mycelia recovery after HS.

### *Po*bZIP3 positively regulates the development of *P. ostreatus*

**FIGURE 10:**
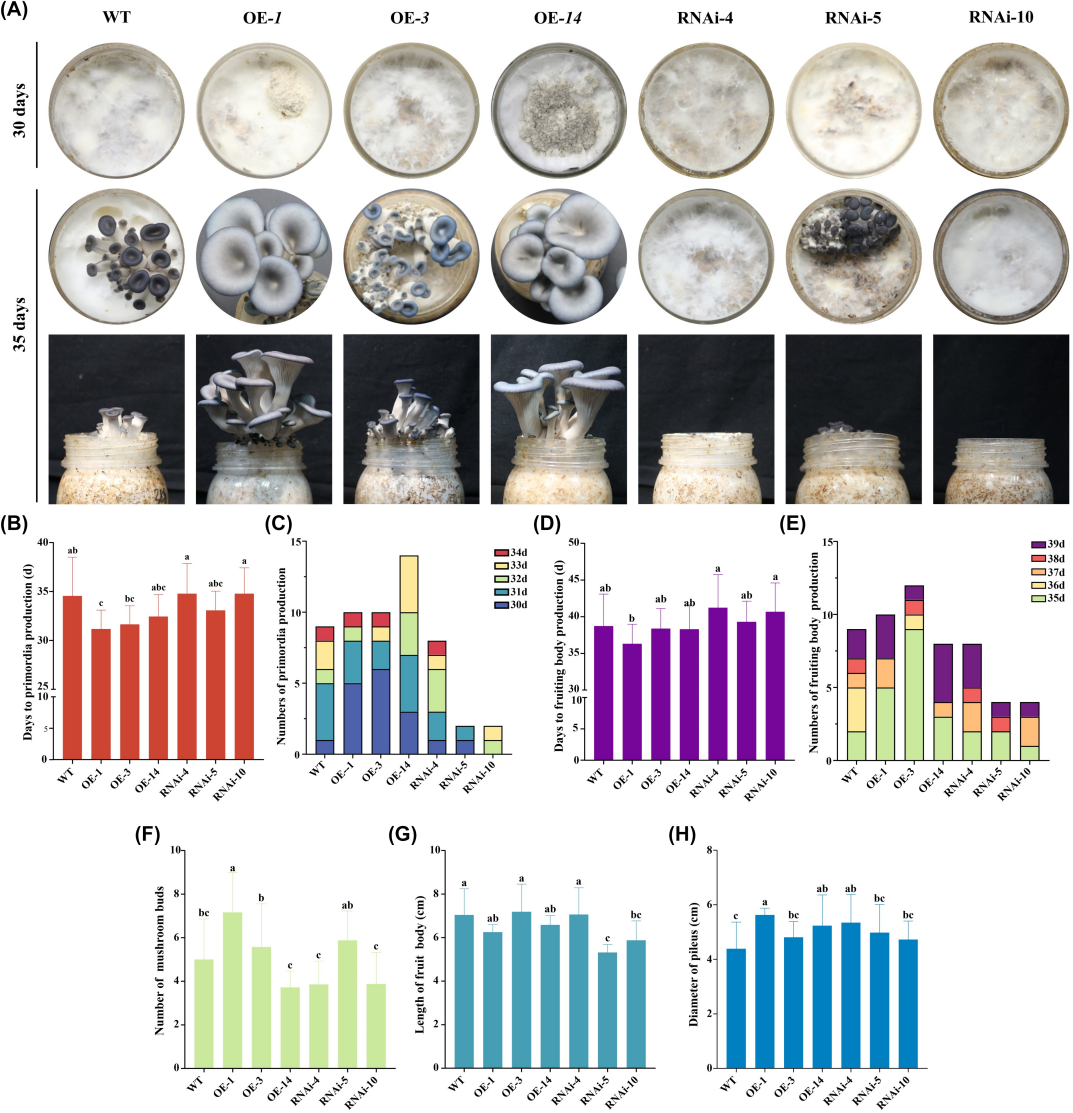
*PobZIP3* reduces the time to primordia emergence and fruiting body production in *Pleurotus ostreatus*. (A) Development of the wild‐type (WT), overexpressing (OE)‐*bZIP3* and RNAi‐*bZIP3* strains. (B–H) Comparison of the (B) primordia formation time, (C) primordia quantity over time, (D) fruiting body production time, (E) fruiting body quantity over time, (F) number of mushroom buds, (G) fruiting body length and (H) pileus diameter of the WT, OE‐*bZIP3* and RNAi‐*bZIP3* strains. The different lower‐case letters indicate statistically significant differences between treatment groups.

To explore the functions of *PobZIP3* during production, the WT, OE‐*bZIP3* and RNAi‐*bZIP3* strains were inoculated to cottonseed hull medium, grown in the dark at 28°C for 26 days, and then transferred to an incubator to stimulate mushroom growth using low temperature and light. Primordia formation time, mushroom bud number and fruiting body morphology were analysed (Figure 10).

The time to primordia formation was not significantly different between the RNAi‐*bZIP3* and the WT strains, averaging 34–35 days; however, the primordia formation time of the OE‐*bZIP3* strains was approximately 31–32 days, which was significantly sooner than the RNAi and WT strains (Figure 10A,B). The time and number of primordia formed in the incubator within 8 days were also statistically analysed; these results showed that the OE strains produced significantly more primordia than the RNAi and WT strains (Figure 10A,C).

The time to fruiting body production of OE‐1 was significantly earlier than RNAi‐4 and RNAi‐10 by approximately 5 days (Figure 10D). The time and number of fruiting bodies produced within 39 days from the start of fruiting were also statistically analysed and the results showed that the OE strains significantly improved the mycelia growth and shortened the fruiting time relative to the RNAi and WT strains (Figure 10A,D,E).

Fruiting body morphology characteristics, such as the number of mushroom buds of each cultivation bottle, length of fruiting body, and diameter of pileus, were also analysed (Figure 10F–H). OE‐1 had significantly more mushroom buds than RNAi‐4 and RNAi‐10; the length of the fruiting body was not significantly different between the OE‐*bZIP3* and the WT strains, which were all significantly longer than the RNAi‐5 strain; and OE‐1 had the largest pileus diameter while the WT strain had the smallest pileus diameter. Although some individual OE and RNAi strains did not differ significantly from WT, the overall agronomic traits of OE were superior to those of RNAi when the OE and RNAi groups were compared. These results indicated that *PobZIP3* positively regulates vegetative growth.

### Changes in *Po*bZIP3 expression lead to transcriptomic alterations

Transcriptome deep sequencing (RNA‐seq) analysis was used to identify the potential heat stress‐related bZIP3‐target genes, using principal component analysis to verify the repeatability among the replicates. Targets were identified as significantly differentially expressed genes (DEGs) when *p* < 0.05 and absolute fold‐change ≥2. Relative to WT, 2243 DEGs were identified in OE‐*bZIP3*, of which 1331 were up‐regulated and 912 were down‐regulated; and 2217 DEGs were identified in RNAi‐*bZIP3*, of which 1321 were up‐regulated and 896 were down‐regulated. GO analysis was used in 288 annotated DEGs to explore the biological processes involving bZIP3, which showed GO term enrichment in pathways including response to stress, antioxidant activity, peptide receptor activity, carbohydrate metabolic process and oxidoreductase activity (Table S3). 8 DEGs related to response to stress encode versatile peroxidase, dual specificity protein kinase kns1, S‐adenosyl‐L‐methionine‐dependent methyltransferase and Cu/Zn‐superoxide‐dismutase; 10 DEGs related to antioxidant activity encode peroxidases (versatile peroxidase, heme‐thiolate peroxidase and DyP‐type peroxidase), glutaredoxin and Cu/Zn‐superoxide‐dismutase; 4 DEGs related to peptide receptor activity encode pheromone receptors (pheromone receptor STE3.3.4 and B mating type pheromone receptor) related to sexual reproduction (Kothe, 2008); 31 DEGs related to carbohydrate metabolic process encode enzymes including glycoside hydrolase, cellobiohydrolase II, polysaccharide lyase family protein and carbohydrate esterase family protein; and the 11 DEGs related to oxidoreductase activity encode enzymes such as peroxidase, laccase and catalase.

## DISCUSSION

TFs coordinate gene expression in cells and the quantity of TFs often determines cellular survival and function (Shelest, 2008). Increasingly, sequenced genomes facilitate the discovery of potential TFs that affect key agronomic traits. Some TFs of *P. ostreatus* were characterized and analysed, 20 *PoMYB* genes were identified and expression analysis of them showed that *PoMYB12*, *PoMYB15* and *PoMYB20* showed extremely high expression levels after heat stress (Wang et al., 2018), then, the function of them was investigated that overexpression of *PoMYB12* and *PoMYB20*, along with RNAi of *PoMYB15*, improved recovery growth post mycelial heat stress, and accelerated the growth and development of *P. ostreatus* (Yuan et al., 2023). And the Zn(II)_2_Cys_6_ zinc cluster‐encoding gene family in *P. ostreatus* also been characterized and analysed, it showed that 13 *PoZCP*s may participate in the development of the heat stress response (Ding et al., 2022). The bZIP TFs, which are prevalent among and exclusive to eukaryotes, are among the largest and most versatile TF families (Wen et al., 2021). They are required for numerous vital biological processes in fungi, including several types of stress responses, mycelial growth, and primary and secondary metabolism (Shin et al., 2020; Wang, Zha, et al., 2020; Wen et al., 2021). Current fungal bZIP research is focused on pathogenic fungi such as *C. chrysosperma*, *M. oryzae* and *F. graminearum* (Kong et al., 2015; Son et al., 2011; Wen et al., 2021); however, there were no reports of bZIP TFs in large edible mushrooms. Therefore, in this study, we conducted comprehensive analyses on the bZIP gene family in *P. ostreatus* to identify the role of bZIP genes throughout the developmental stages and heat stress response, which will provide insights into the function of *P. ostreatus* bZIP genes.

Herein, we identified and systematically analysed 11 bZIP genes, accounting for 3.97% of all *P. ostreatus* TFs (Hou et al., 2020). Notably, both the number and proportion of bZIPs in *P. ostreatus* were less than the average for *Agaricomycotina* (14.5, 5%) (Todd et al., 2014), suggesting that the number of bZIP genes was reduced during evolution relative to other fungi. Additionally, the phylogenetic analysis of bZIP genes in *P. ostreatus* and 9 other fungal species showed that the *PobZIP*s were difficult to cluster into a broad category with other bZIPs (Figure 4), suggesting that *PobZIP*s developed late compared to the differentiation of *Agaricomycotina* and that the underlying molecular mechanisms may therefore differ. From the genetic structure analysis, we found that the number of introns varied among the *PobZIP*s (Table 1 and Figure 3C), ranging from 0 to 12 introns, whereas previous studies have shown that the bZIPs from *C. minitans*, switchgrass, poplar, *Chenopodium quinoa* and Chinese jujube contain 6, 14, 12, 11 and 11 introns, respectively (Li et al., 2020; Wang, Wang, et al., 2020; Xu et al., 2020; Zhang et al., 2020; Zhao et al., 2021), while the bZIPs of the notorious pathogenic fungus *C. chrysosperma* only contain up to three introns (Wen et al., 2021). Studies have shown that, after segment replication events, the loss of introns occurs at a faster rate than the gain of introns (Nuruzzaman et al., 2010), and our results suggest the occurrence of a putative *P. ostreatus* gene duplication event between the plant and *C. chrysosperma*. The *Po*bZIPs all contained a core bZIP domain that can preferentially bind the sequence of their downstream genes through specific cis‐acting elements (Wang, Zha, et al., 2020), and the other domains may confer a diversity of functions to the bZIP family (Jakoby et al., 2002; Nijhawan et al., 2007; Yasunari Fujita, 2013).

bZIP proteins participate in the regulation of signalling and abiotic/biotic stimuli responses, including hypersalinity, drought, osmotic stresses, temperature stresses and pathogen defence (Baloglu et al., 2014; Li et al., 2015; Nuruzzaman et al., 2010). Several predicted cis‐acting elements in the *PobZIP* promoter regions are associated with hormone responsiveness, growth and development, stress responsiveness, metabolism regulation and transcriptional regulation. Gene expression analysis can often provide useful predictions for the function of multi‐gene families (Li et al., 2020). Thus, we explored expression patterns of the *PobZIP* genes under heat stress; according to our expression profile data, the expression levels of some family members changed significantly with the duration of heat stress, implying their involvement in the heat stress response. The bZIP TFs have also been shown to perform critical roles in organism growth and development. In *Cannabis sativa L*, the *CsbZIP*s exhibited increased expression in flowers and bracts, indicating that *CsbZIP*s are essential for the growth and development (Lu et al., 2022). Moreover, DKM—a member of the *Arabidopsis thaliana* bZIP TF family—can negatively modulate *Arabidopsis* growth and reproductive development (Lozano‐Sotomayor et al., 2016). The expression patterns of the *PobZIP*s varied throughout the different developmental stages, many of which were up‐ or down‐regulated at specific stages of development (Figure 7). Further studies should be conducted to investigate the functions of developmental stage‐specific genes and genes that are regulated during substratum formation.

As a promoter, bZIP can form homo‐ or heterodimers and regulate downstream‐related genes by binding to cis‐acting elements such as G‐box, C‐box, ABRE, LTRE and BoxII (Riechmann et al., 2000; Z et al., 2014). Previous studies have shown that bZIP can interact with HSPs (Ma et al., 2018; Z et al., 2014); since we previously showed that *HSP100* participates in the *P. ostreatus* heat stress response—*PoHSP100* expression was up to 15 times higher under heat stress relative to controls (Zou et al., 2018)—we used yeast two‐hybrid assays to screen HSP100 for potential interactions with bZIPs and found that only *Po*bZIP3 interacted with HSP100. The GST‐Pull‐down assay and EMSA were then performed, which revealed that *Po*bZIP3 can combine with G‐box and C‐box sequences to regulate *PoHSP100* transcription, which suggests that *Po*bZIP3 enhances resistance to heat stress in mushrooms by regulating the expression of *PoHSP100*.

The bZIP TF family has numerous functions and is involved in the abiotic stress response in a variety of fungi. In addition, it also participates in primary and secondary metabolic reactions and affects the growth and development of fungi. Knockout experiments on the 12 bZIP TFs of *Alternaria alternata* resulted in decreased growth rate after the elimination of *bZIP17*, *bZIP22* and *bZIP24*; significantly decreased air‐exposed mycelia after the elimination of *bZIPI9* and *bZIP21*; and lack of spore production and significantly reduced sensitivity to oxidative stress after the elimination of *bZIP21* (Gai et al., 2022). The sensitivity of *C. minitans* to H_2_O_2_ was significantly enhanced after *CmbZIP16* was knocked out, suggesting that CMbZIP16 is critical for the oxidative stress response (Xu et al., 2020). After silencing the *Aspergillus parasiticus* bZIP TF AtfB, the mycelium appeared white and villous, the number of conidium decreased and the aflatoxin enzyme activity and accumulation decreased. Moreover, the expression levels of many genes related to secondary metabolism and conidium development decreased, altogether indicating that AtfB helps to regulate growth, development and secondary metabolism (Wee et al., 2017). Our results showed that overexpressing *PobZIP3* positively regulated both the tolerance to heat stress and the ability to recover from heat stress in *P. ostreatus* mycelium. In fruiting development, overexpression of *PobZIP3* resulted in significantly earlier transformant progenitor formation than WT, while interfering transformant progenitor formation was significantly delayed. Based on previous studies, bZIP TFs are functionally extensive, affecting growth and development through a variety of pathways. In *C. chrysosperma*, *CcbZIP05* and *CcbZIP23* have been shown to be associated with fungal growth, while *CcbZIP23* was involved in the regulation of conidial production and colour production (Wen et al., 2021).

**FIGURE 11:**
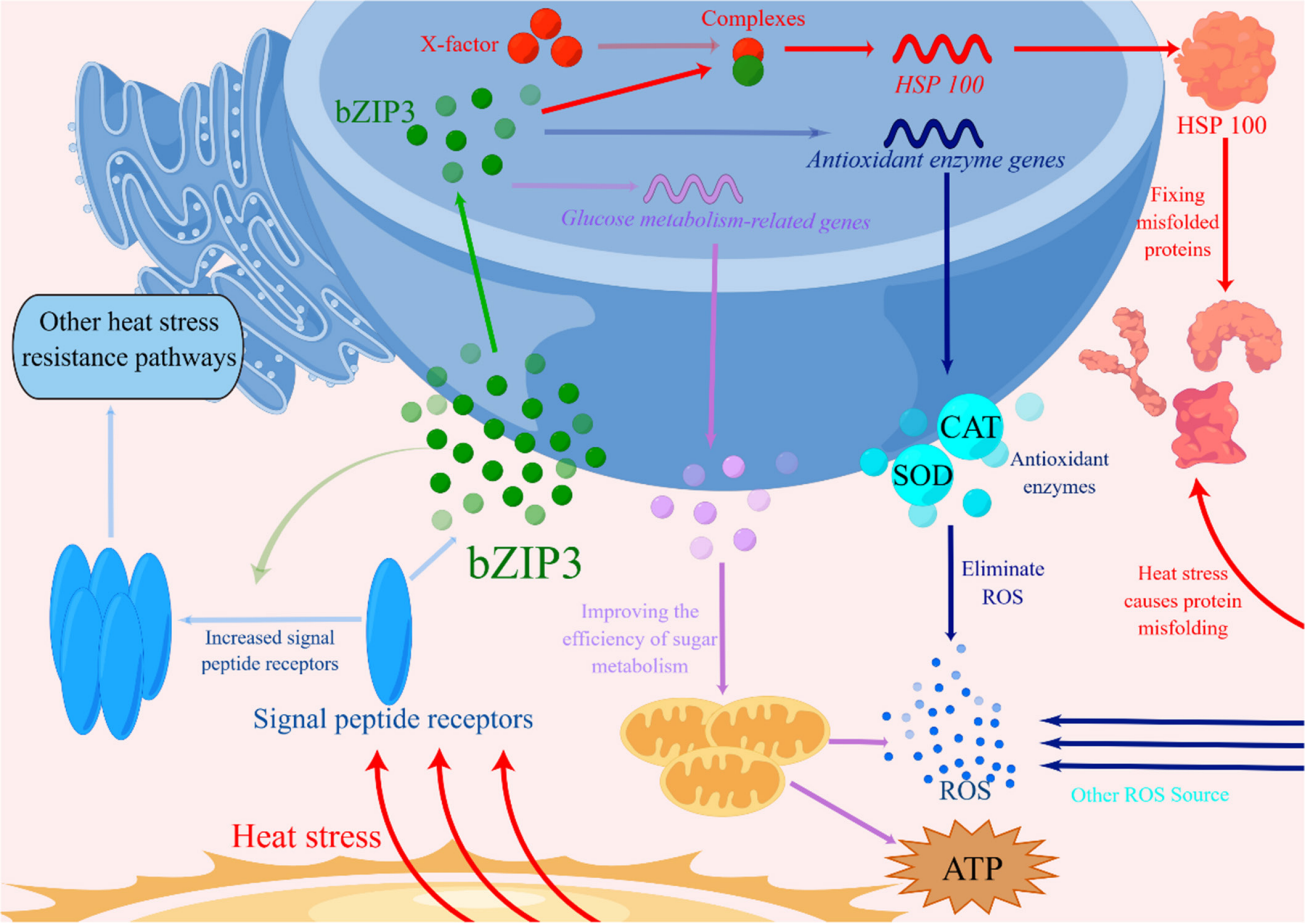
Hypothetical mechanism of *Po*bZIP3 in *Pleurotus ostreatus* during the heat stress response. The translucent arrow points to the presumed mechanism of *Po*bZIP3.

bZIP is widely involved in various metabolic processes and signal transduction pathways in living organisms. In wheat, *Ta*bZIP15 interacted with *Ta*ENO‐b, which catalysed the reversible dehydration of 2‐phospho‐D‐glycerol to phosphoenolpyruvate, a reaction that is part of the glycolytic and gluconeogenic pathways (Bi et al., 2021). Overexpression of sweet potato *IbbZIP1* in *Arabidopsis* resulted in significant up‐regulation of reactive oxygen scavenging system‐related genes, increased superoxide dismutase activity and decreased H_2_O_2_ concentration (Kang et al., 2019). Overexpression of blueberry VabZIP12 in *Arabidopsis* led to significantly up‐regulated enzymatic antioxidant activity and expression of related genes, thereby enhancing its salt stress tolerance (Qu et al., 2022). In our study, the transcriptome data from WT, OE‐*PobZIP* and RNAi‐*PobZIP* strains under 40°C heat stress showed that DEGs were mainly enriched in catalytic activity, carbohydrate metabolic processes, extracellular regions, antioxidant activity, stress response, peroxidase activity, pheromone receptor activity and oxidoreductase activity, with the most enriched genes mainly involved in sugar metabolic pathways, antioxidant enzyme activity and sexual reproduction processes. This result may indicate that, under heat stress, *PobZIP3* regulates intracellular ROS homeostasis by regulating the activity of intracellular antioxidant enzymes, achieves cellular homeostasis by regulating intracellular glucose metabolism to regulate intracellular energy supply, and can also respond to heat stress by affecting cellular reproduction (Figure 11).

By analysing the transcriptome results, we also discovered an interesting phenomenon, namely, that *PoHSP100* expression was not significantly altered by the overexpression or silencing of *PobZIP3*; however, since our experimental evidence suggested that the two interacted with each other at the protein level, we hypothesized that the interaction between *Po*bZIP3 and *Po*HSP100 involves another acting factor X that forms a dimer with *Po*bZIP3 to act jointly on *Po*HSP100 (Figure 11). Previous studies have also found that bZIP TFs form dimers that jointly affect the expression of downstream genes. In apples, *Md*bZIP80 can form a dimer with *Md*bZIP2 or *Md*bZIP39 to act on the *MdIPT5b* promoter region, directly repressing gene transcription and negatively regulating drought tolerance, whereas they are unable to repress *MdIPT5b* individually (Feng et al., 2021). Additionally, heat stress in *Arabidopsis* induces dephosphorylation of bZIP18 and bZIP52, causing them to shift from the cytoplasm to the nucleus where they form dimers that jointly regulate the expression of downstream genes and affect several metabolic pathways such as energy metabolism and translation (Wiese et al., 2021).

In this study, firstly, the *PobZIP* genes were identified and analysed. Secondly, expression pattern analysis during heat stress treatments and different development stages revealed that *PobZIP*s participated in the heat stress tolerance pathways. Thirdly, results from yeast two‐hybrid assays, GST‐Pull‐down assays and EMSA confirmed the interaction between *Po*bZIP3 and *Po*HSP100. Finally, functional analysis showed that *Po*bZIP3 could relieve heat stress‐induced damage to mycelium, enhance its growth recovery ability and reduce the time to protoplast emergence and fruiting production.

## CONCLUSION

In summary, bZIP TFs were identified on a *P. ostreatus* genome‐wide level. The gene structures, chromosomal locations, phylogenetic relationships and stress‐ and hormone‐related cis‐acting elements were also analysed. Furthermore, *PobZIP* expression patterns under heat stress and during different development stages were analysed using RNA‐seq and RT‐qPCR techniques. The *Po*bZIP3–*Po*HSP100 interaction showed that *Po*bZIP3 is vital to the heat stress response. *PobZIP3* overexpression enhanced the heat tolerance of the mycelium and brought forward the harvest time, whereas RNAi had the opposite result. RNA‐seq results showed that *Po*bZIP3 may be involved in sugar metabolic pathways, antioxidant enzyme activity and sexual reproduction processes of *P. ostreatus*. However, further experimental work is required to determine the more specific function of *Po*bZIP3 in oyster mushroom.

## AUTHOR CONTRIBUTIONS

**Yuanyuan Zhou:** Data curation (lead); formal analysis (lead); methodology (lead); project administration (equal); resources (lead); software (lead); validation (lead); visualization (lead); writing – original draft (lead); writing – review and editing (equal). **Zihao Li:** Investigation (equal); methodology (equal). **Congtao Xu:** Investigation (equal); methodology (equal). **Jinlong Pan:** Investigation (equal); methodology (equal). **Haikang Li:** Writing – original draft (equal); writing – review and editing (equal). **Yi Zhou:** Data curation (equal); formal analysis (equal). **Yajie Zou:** Funding acquisition (equal); project administration (equal); writing – review and editing (equal).

## CONFLICT OF INTEREST STATEMENT

The authors have no competing interests to declare.

## Supporting information


Table S1

Table S2

Table S3

## Data Availability

The data used to support the study findings are included within the article.
